# Correlation between intestinal microbiota and urolithin metabolism in a human walnut dietary intervention

**DOI:** 10.1186/s12866-024-03626-5

**Published:** 2024-11-15

**Authors:** Huijia Liu, John W. Birk, Anthony A. Provatas, Haleh Vaziri, Nuoxi Fan, Daniel W. Rosenberg, Raad Z. Gharaibeh, Christian Jobin

**Affiliations:** 1 Department of Medicine, University of Florida College of Medicine, Gainesville, FL USA; 2 Division of Gastroenterology, University of Connecticut, Farmington, CT USA; 3 Center for Environmental Sciences and Engineering, University of Connecticut, Storrs, CT USA; 4 School of Medicine, University of Connecticut, Farmington, CT USA; 5 Department of Molecular Genetics and Microbiology, University of Florida, Gainesville, FL USA; 6 Department of Infectious Diseases and Immunology, University of Florida College of Medicine, Gainesville, FL USA; 7 Department of Anatomy and Cell Biology, University of Florida College of Medicine, Gainesville, FL USA

**Keywords:** Human gut microbiota, Urolithin, 16S RNA gene sequencing, Clinical study, Walnut

## Abstract

This study is to investigate the relationship between the intestinal microbiota and urine levels of the ellagic acid-derived polyphenols, the urolithins, in a cohort of subjects following a three-week walnut dietary intervention. We longitudinally collected fecal and urine samples from 39 subjects before and after walnut consumption (2 oz per day for 21 days). 16S RNA gene sequencing was performed on fecal DNA to study the association between microbiota composition and the levels of nine urolithin metabolites, which were measured using UHPLC/Q-TOF–MS/MS. Fecal microbial composition was found to be significantly different between pre- and post-walnut intervention (beta diversity, FDR-*p* = 0.018; alpha diversity, *p* = 0.018). *Roseburia*, *Rothia*, *Parasutterella*, *Lachnospiraceae* UCG-004, *Butyricicoccus*, *Bilophila*, *Eubacterium eligens*, *Lachnospiraceae* UCG-001, *Gordonibacter*, *Paraprevotella*, *Lachnospira*, *Ruminococcus torques*, and *Sutterella* were identified as the 13 most significantly enriched genera after daily intake of walnuts. We observed 26 genera that were significantly associated with 7 urolithin metabolites, with 22 genera positively correlating after walnut supplementation (FDR-*p* ≤ 0.05). PICRUSt analysis showed that several inferred KEGG orthologs were associated with 4 urolithin metabolites after walnut intake. In this study, we found that walnut supplementation altered urolithin metabolites, which associates with specific changes in bacterial taxa and inferred functional contents.

**Supplementary Information:**

The online version contains supplementary material available at 10.1186/s12866-024-03626-5.

## Introduction

Walnuts are a rich source of the ellagitannins, complex plant-based polyphenolic compounds, that are slowly hydrolyzed in the GI tract to release free ellagic acid (EA) [1]. Ellagitannins have been studied for their wide range of antioxidant and anti-inflammatory properties [2]. The gut microbiota converts ellagic acid into the diverse panel of urolithins that are increasingly recognized as an important group of molecules for human health [3, 4]. Earlier studies have examined the effects of walnuts in several mouse models of intestinal disease [4–6]. For example, using the colon carcinogen, azoxymethane (AOM), our laboratory [7] tested the effects of walnuts added to a Total Western Diet (TWD), formulated to recapitulate the typical American intake of macro- and micronutrients and fat from multiple sources, matching patterns of fat consumption as reported by NHANES [8]. We found a significant reduction in tumors in mice consuming 7% walnuts [7]. We identified a microbial composition directly associated with colorectal cancer (CRC) suppression, similar to those reported for ulcerative colitis [5] and inflammation-associated CRC [9]. These findings suggest that walnut dietary supplementation, in part through bacteria-derived production of urolithins, may elicit preventive effects on CRC.

Although there are many urolithin metabolites, urolithin A and iso-urolithin A are of particular interest for their potent anti-cancer, anti-inflammatory, and prebiotic activities [3, 10, 11]. Urolithin A has a wide range of effects on cancer signaling and inflammatory pathways [12]. An important property of urolithin A is its ability to cause an overall improvement in mitochondrial health, driven in part by its induced clearing and recycling of dysfunctional mitochondria via mitophagy [13]. In fact, the urolithin A-induced effects on mitophagy, particularly in immune cells, may play an important role in its ability to affect tumorigenesis. Urolithin A also acts as an anti-inflammatory agent, first shown by its ability to inhibit COX-2 in a Dextran Sulfate Sodium (DSS) induced colitis model [14] and a reduction in proinflammatory cytokines (IL-1b, IL-6, TNF-a) [15].

However, preclinical cancer prevention studies with urolithin A have been limited. The first study of its kind provided urolithin A to C57BL/6 mice and detected high levels of the compound in prostate, intestine, and colon, peaking at 4 h after oral administration [16]. In addition, urolithin A delivered as pomegranate extract also suppressed prostate cancer cell growth in a tumor explant model [16]. Based on its ability to target PI3K/AKT/mTOR signaling [17], urolithin A was also tested in a pancreatic ductal carcinoma (PDAC) xenograft mouse model. Reduced growth of tumors was observed, associated with reprogramming of the tumor microenvironment, including reduced levels of myeloid-derived suppressor cells, tumor-associated macrophages, and T-regulatory cells [17].

The production of urolithins in individuals can vary substantially, likely due to differences in the individual microbiome [18–20]. Here, we investigated the impact of walnut supplementation on microbiota composition and inferred microbial functional contents in healthy adult subjects scheduled for a screening or diagnostic colonoscopy, and whether these changes may be correlated with the formation of specific urolithin metabolites. Our data suggest that the intestinal microbiota and the levels of urolithin metabolites are modified by walnut supplementation, with specific changes in microbial genera and inferred functional contents associated with certain urolithin metabolites.

## Materials and methods

### Study population and urolithin metabolites

The cohort used in this study was previously described [21]. In brief, 39 healthy adult subjects completed the clinical study, consisting of walnut supplementation (2 oz/day whole-peeled) for a period of 21 days, with fecal and urine samples collected before and after the dietary intervention. The majority of our study participants are white (65%), followed by Asian (20%) and African American (12.5%) (Supplementary Table 5). During the study, participants were given sealed packets containing 1 oz of whole-peeled walnuts and were instructed to consume two packets daily—one in the morning and one in the evening. They were also asked to avoid foods and beverages high in ellagitannins, such as berries, pomegranates, grapes, pecans, almonds, and oak-aged beverages, starting one week before walnut supplementation and continuing throughout the 3-week walnut consumption period. This study was approved by the UConn Health Institutional Review Board (IRB # 19-121JS-1) at UConn Health. All participants provided written informed consent prior to enrollment in the study. Urine samples were analyzed by ultra-high performance liquid chromatography with quadrupole time-of-flight mass spectrometry (UHPLC/Q-TOF–MS/MS), an analytic pipeline we designed [22] for measuring nine urolithin metabolites (urolithin A, iso-urolithin A, urolithin B, urolithin C, urolithin D, urolithin E, urolithin M5, urolithin M6, and urolithin M7).

### Fecal collection, DNA extraction, and 16S RNA gene sequencing

Stool samples before and after walnut diet were self-collected from each subject using a pre-labeled, self-sampling stool collection kit with detailed instructions for collection, storage, and prepaid shipment as described previously [21]. Human fecal samples were extracted using the DNeasy® 96 PowerSoil® Pro QIAcube® HT (QIAGEN). Briefly, approximately 200 mg of each tube of feces was promptly added to a PowerBead ProPlate and stool was homogenized through the TissueLyser II (QIAGEN), to which 800 µL of CD1 was added and a standard kit procedure was used to isolate the DNA. NanoDrop™ Eight Spectrophotometer was used to evaluate DNA concentration and quality. All samples were then assigned to extraction batches, with four randomly negative controls included (extraction blank). Additionally, positive PCR controls were used, including ZymoBIOMICS microbial community and microbial community DNA standards. Following total fecal DNA extraction, the 585-bp of 16S RNA V3-V4 hypervariable region was amplified using barcoded primer pairs 341F (5’ = -CCTACGGGNGGCWGCAG-3’ =) and 785R (5’ = -GACTACHVGGGTATCTAATCC-3’ =) with universal Illumina paired-end adapter sequences and unique individual 4–6-nucleotide barcodes for multiplex PCR sequencing. The 20 µL PCR reaction was composed of 11.3 µL of molecular biology grade water, 2 µL of 10X buffer, 1.2 µL of MgCl_2_, 0.4 µL of dNTPs, 1 µL of each forward and reverse primer at 5 µM concentration (final concentration of 0.25 µM), 0.1 µL of Invitrogen Taq DNA polymerase (ThermoFisher Scientific) and 3 µL of 5 ng/µL genomic DNA from each sample, including Zymo, standard, and water control. PCR was performed in a standard thermocycler with a 2 min preincubation step at 94 °C, followed by 20 cycles of 20 s at 94 °C, 30 s at 56°C, and 45 s at 72°C, with a final extension of 7 min at 72°C. 5 µL PCR products were visualized on a 2% agarose DNA gel. PCR products were then quantified by qPCR though KAPA Library Quantification Kit (KAPA Biosystems). 5 ng of samples were pooled in a sequencing library and purified by AMPure XP kit (Beckman Coulter) to remove unincorporated dNTPs, primers, primer dimers, salts and other contaminants. KAPA Kit were then used for quantification of pooling samples and samples sequenced on Illumina MiSeq. One sample from timepoint A failed sequencing and was excluded.

### Analysis of 16S RNA gene sequences

De-multiplexed reads were fed to the DADA2 pipeline [23] for pair-merging, primer sequence removal, quality filtering, correction of Illumina amplicon sequencing errors, and de-replication, followed by amplicon sequence variants (ASVs) generation and chimera removal. ASVs were checked for possible contamination using the decontam R package [24] using the prevalence method, and no contaminants were detected. We then removed any sequence that was classified as non-bacterial and all singleton ASVs. This resulted in a total of 8,428,349 reads, with an average of 109,459 reads per sample (min = 14,814; max = 176,891) and 4,529 ASVs. Taxonomic classification was performed using DADA2 assignTaxonomy and addSpecies functions utilizing SILVA reference dataset (v. 138.1).

Principal Coordinate Analysis (PCoA) was generated using the phyloseq package [25] from Bray–Curtis dissimilarity matrix after count normalization and log_10_ transformation and described previously in Arthur et.al [26] and McCafferty et al. [27]. Alpha diversity (Shannon diversity index) was calculated using the same package after rarefying the counts to the minimum count of all samples (14,814 reads).

Differences in the microbial community structure (beta diversity) were tested using permutational multivariate analysis of variance (PERMANOVA) using the vegan R package command adonis (version 2.6) with 1000 permutations and stratified on subject ID to account for multiple measures from the same subject. Differences in the alpha diversity were tested using Linear Mixed-Effects Model (lme) in R nlme package (v.3.1–163) with subject set as a random effect to account for multiple measures from the same subject. Differential taxa abundance analysis was performed using MaAsLin2 R package [28] with subject set as a random effect. In addition to timepoint, and using the approaches described above (PERMANOVA, lme, and MaAsLin2), we evaluated the subjects for the following variables: age, urolithin producer status, sex, ethnicity, race, BMI, smoking status, alcohol consumption status, drug usage, diet, GI history, laxative usage, probiotics usage, other GI, other health conditions, disease status, and number of polyps detected during colonoscopy to make sure the detected differences in the alpha, beta and taxa analyses are not due to these variables.

KEGG Ortholog (KO) functional profiles of microbial communities were predicted from the 16S RNA gene sequences using phylotypic investigation of communities by reconstruction of unobserved states (PICRUSt2) [29]. ASVs were used for PICRUSt2 predictions according to PICRUSt2 developers’ guidelines.

### Analysis of urolithin metabolites

Urolithin metabolite values were normalized and log_10_-transformed as described above. Principal Component Analysis (PCA) was performed on the normalized and log_10_-transformed values using R prcomp function. We used Linear Mixed-Effects Model (lme) in R nlme package (v.3.1–163) with subject set as a random effect to account for multiple measures from the same subject to test for differences between the two timepoints. Spearman correlation analysis was performed to identify gut microbiota genera associated with different urolithin metabolites through R cor.test function. Correlation *P*-values were then FDR corrected and only those with FDR adjusted *P*-value ≤ 0.05 were considered.

Correlations between taxa abundance, KEGG Orthologs, and urolithin metabolites were done using Spearman correlation through R cor.test function. Correlation *P*-values were then FDR corrected sing R p.adjust function employing the method of Benjamini and Hochberg [30] and only those with an FDR adjusted *P*-value < 0.01 were considered for further analysis.

## Results

### Walnut consumption alters gut microbiota composition

Overall, we observed a significant increase in total urolithin metabolites after walnut intervention (Supplementary Fig. 1A, *p* = 0.00003). The PCA revealed significant clustering difference between before (timepoint A) and after (timepoint B) walnut supplementation, based on the levels of nine urolithins (Supplementary Fig. 1B, *p* = 0.0000131). Analysis of individual urolithin metabolites in the study cohort demonstrated that walnut consumption significantly augmented the levels of total urolithins, urolithin A, and iso-urolithin A present in the urine compared to baseline (Supplementary Fig. 1C).

**Fig. 1:**
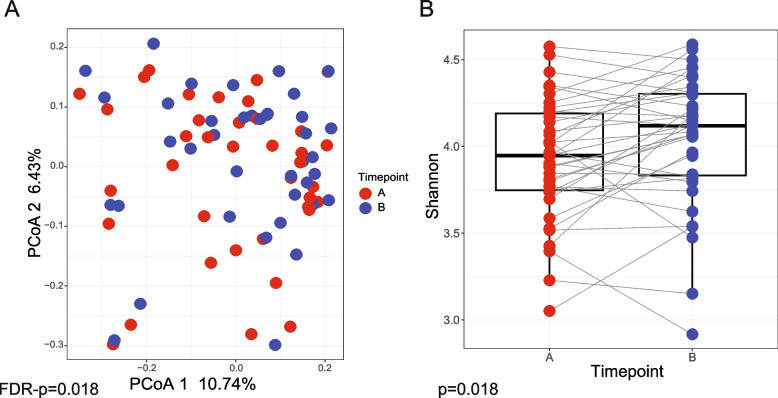
The difference in gut microbiota composition before and after walnut consumption. **A** Principal coordinate analysis (PCoA) showing beta diversity measured by Bray–Curtis dissimilarity between timepoints (*p* = 0.001, FDR-*p* = 0.018). **B** Alpha-diversity measured by the Shannon index for subjects from timepoint A and timepoint B (*p* = 0.018, FDR-*p* = 0.159). Samples before walnut dietary intervention represent timepoint A (*n* = 38), and samples after walnut dietary intervention represent timepoint B (*n* = 39). *P* values < 0.05 were considered statistically significant

**Fig. 2:**
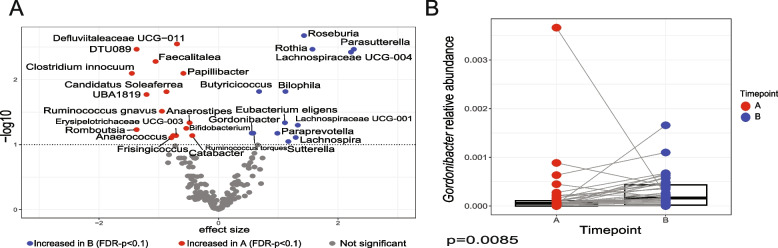
Identification of differentially enriched microbial genera between before and after walnut intake. **A** Volcano plot showing differences in the gut microbiome genera between timepoints (FDR-*p* < 0.1). See Supplemental Table 1 for full list. **B** Box plot showing *Gordonibacter* relative abundance at both timepoints (*p* = 0.0085, FDR-*p* = 0.067). Samples before walnut dietary intervention represent timepoint A (*n* = 38), and samples after walnut dietary intervention represent timepoint B (*n* = 39). *P* values < 0.05 were considered statistically significant

We next studied the effects of walnut supplementation on gut microbiota, with the goal of correlating bacteria with urolithin profiles. To do this, we analyzed gut microbial composition before and after walnut supplementation using 16S RNA gene sequencing. Principal coordinates analysis (PCoA) showed significant difference in microbiota beta diversity (Fig. 1A, FDR-*p* = 0.018, and Supplementary Fig. 2), as well as a change in Shannon's alpha-diversity (Fig. 1B, *p* = 0.018, FDR-*p* = 0.159) after walnut consumption. These differences were independent from other cohort variables, such as age, sex, urolithin producers, and ethnicity, as described (Supplementary Table 1) [21]. We used MaAsLin2 to examine the differences in the relative abundance of genera detected in our 16S RNA gene analysis after walnut supplementation (Fig. 2A). We detected 15 genera (*Defluviitaleaceae* UCG-011, DTU089, *Faecalitalea*, *Clostridium innocuum*, *Papilibacter*, *Candidatus Soleaferrea*, UBA1819, *Ruminococcus gnavus*, *Anaerostipes*, *Bifidobacterium*, *Romboutsia*, *Anaerococcus*, *Erysipelotrichacease* UCG-003, *Frisingicoccus*, and *Catabacter*) that were significantly enriched before walnut consumption, whereas 13 genera (*Roseburia*, *Rothia*, *Parasutterella*, *Lachnospiraceae* UCG-004, *Butyricicoccus*, *Bilophila*, *Eubacterium eligens*, *Lachnospiraceae* UCG-001, *Gordonibacter*, *Paraprevotella*, *Lachnospira*, *Ruminococcus torques*, and *Sutterella*) were significantly enriched after daily intake of walnut (Fig. 2A, FDR-*p* < 0.1). Importantly, we found that the relative abundance of *Gordonibacter* was increased after walnut consumption (Fig. 2B, *p* = 0.0085, FDR-*p* = 0.067). This genus is able to produce urolithin A and iso-urolithin A from ellagic acid in vitro [31].

### Walnut supplementation modifies inferred microbial gene pathways

**Fig. 3:**
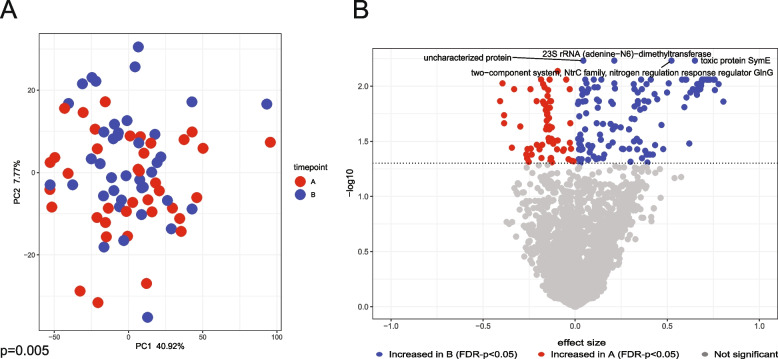
The difference in inferred functional contents before and after walnut consumption. **A** PCA demonstrating the difference in KEGG orthologs between timepoints (*p* = 0.005). **B** Volcano plot of KEGG orthologs at timepoint A and timepoint B (*p* < 0.01). See Supplemental Table 2 for full list. Samples before walnut dietary intervention represent timepoint A (*n* = 38), and samples after walnut dietary intervention represent timepoint B (*n* = 39). *P* values < 0.05 were considered statistically significant

Next, we analyzed the data using Phylogenetic Investigation of Communities by Reconstruction of Unobserved States (PICRUSt2), which infer functional contents from 16S RNA gene sequencing data [29]. This analysis demonstrated significant difference between before (timepoint A) and after (timepoint B) walnut supplementation samples based on their PICRUSt2 inferred KEGG orthologs (Fig. 3A, *p* = 0.005). We detected 45 orthologs: 39 increased after walnut supplementation (timepoint B), and 6 increased before walnut supplementation (timepoint A) (Fig. 3B, *p* < 0.01, Supplementary Table 2).

### Different microbial genera associated with urolithin metabolites

**Fig. 4:**
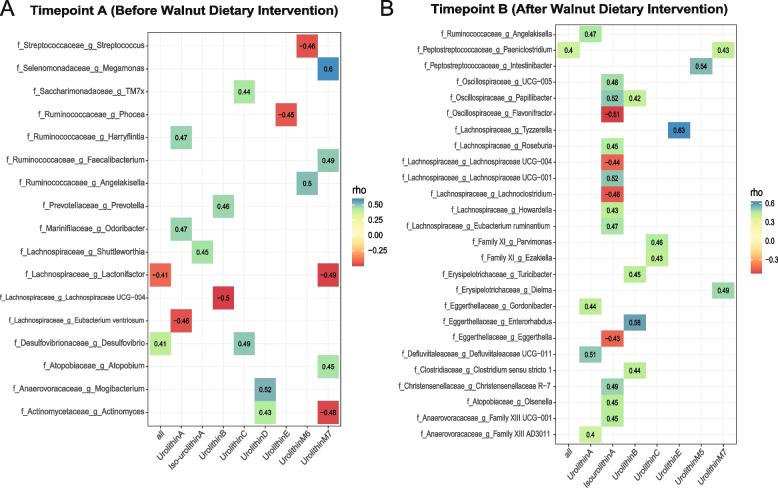
Association between genera abundances and measured urolithin levels before and after walnut supplementation. **A** The correlation heatmap of gut microbiota genera and different urolithin metabolites (FDR-*p* ≤ 0.05, rho > 0.4 or rho < -0.4). Spearman's correlations between 17 enriched relative abundance of bacterial genera and 8 urolithin metabolites at timepoint A. **B** Correlation between gut microbiota genera and different urolithin metabolites (FDR-*p* ≤ 0.05, rho > 0.4 or rho < -0.4). Spearman's correlations between 26 enriched relative abundance of bacterial genera and 7 urolithin metabolites at timepoint B. Blue represents positive correlations, and red represents negative correlations. See Supplemental Table 3 for full list

To identify specific bacteria associated with urolithin metabolites, we performed a correlation analysis between relative abundance of microbial genera and urolithin metabolite levels. Many bacteria were associated with urolithin metabolites along the walnut supplementation timeline (Fig. 4, FDR-*p* ≤ 0.05, rho > 0.4 or rho < -0.4). We observed 26 genera that were significantly associated with 7 urolithin metabolites after walnut intake (Fig. 4B, FDR-*p* ≤ 0.05). Overall, 7 negative correlations were observed before walnut introduction, while only 4 were noted after walnut supplementation, especially iso-urolithin A (Fig. 4A and B, FDR-*p* ≤ 0.05). Interestingly, *Lachnospiraceae Shuttleworthia* was the only genus positively correlated with iso-urolithin A at baseline (Fig. 4A, FDR-*p* ≤ 0.05) whereas 13 genera correlated (9 positives and 4 negatives) with production of this metabolite after walnut supplementation (Fig. 4B, FDR-*p* ≤ 0.05). In addition, *Marinifilaceae Odoribacter*, *Ruminococcaceae Harryflintia*, and Lachnospiraceae Eubacterium ventriosum were mainly associated with production of the urolithin A metabolite before walnut intake (Fig. 4A, FDR-*p* ≤ 0.05), whereas *Eggerthellaceae Gordonibacter*, *Ruminococcaceae Angelakisella*, *Defluviitaleaceae Defluviitaleaceae UCG − 011*, and *Anaerovoracaceae Family XIII AD3011* were associated with its production after supplementation (Fig. 4B, FDR-*p* ≤ 0.05).

### Association between inferred microbial functions and urolithin metabolism

**Fig. 5:**
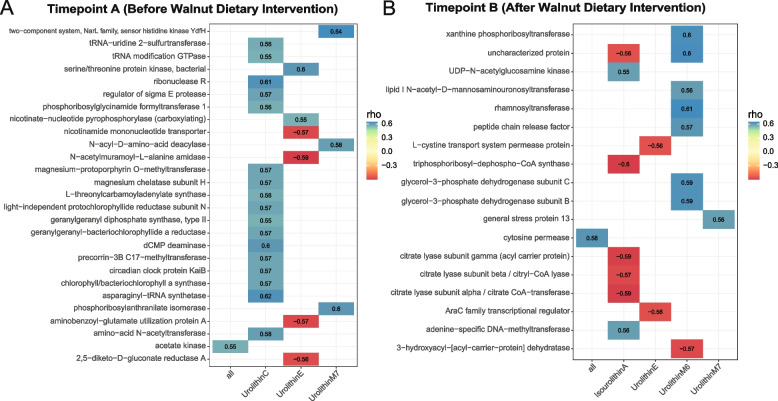
Association between inferred functional contents and urolithin metabolites before and after walnut supplementation. **A** Correlation between KEGG orthologs and urolithin metabolites at timepoint A (FDR-*p* < 0.01, rho > 0.55 or rho < -0.55) and (**B**) timepoint B (FDR-*p* < 0.01, rho > 0.55 or rho < -0.55). Blue represents positive correlations, and red represents negative correlations. See Supplemental Table 4 for full list

To examine the relationship between inferred microbial functional content and urolithin metabolites before and after walnut supplementation, we compared the inferred PICRUSt KEGG orthologs associated with urolithin metabolites by correlation analysis. Compared to healthy adults before walnut supplementation, a lower number of microbial orthologs were positively associated with the formation of 4 urolithin metabolites after walnut intake, and higher number of orthologs showed a negative correlation with respect to iso-urolithin A and urolithin E (Fig. 5, FDR-*p* < 0.01, rho > 0.55 or rho < -0.55). For samples before walnut supplementation, positive associations between KEGG orthologs and urolithin metabolites (17 orthologs of urolithin C, and 3 orthologs of urolithin M7) accounted for most of the significant correlations (Fig. 5A, FDR-*p* < 0.01). Importantly, 7 orthologs correlated (2 positively and 5 negatively) with iso-urolithin A after walnut supplementation, while no orthologs were found to be significantly correlated with iso-urolithin A at baseline (Fig. 5B, FDR-*p* < 0.01). Overall, our data showed that walnut supplementation leads to specific bacterial changes, which correlates with distinct functional profiles of urolithin production.

## Discussion

Incorporating polyphenol-rich foods, such as walnut, can modulate gut microbiota by increasing the abundance of beneficial bacteria like *Phascolarctobacterium*, *Coprococcus*, and *Anaerostipes* [32]. These bacteria contribute to the production of short-chain fatty acids, such as acetate and propionate, which are important for gut health and have anti-inflammatory and metabolic benefits [33, 34]. This modulation of the gut microbiome through polyphenol intake supports overall health by promoting a more diverse and balanced microbial community. Our longitudinal study of a healthy United States cohort revealed that human gut microbial composition was significantly different following walnut intervention (2 oz/day for 21 days). Notably, significantly enriched genera and inferred microbial functions were different between timepoints and were found to be associated with different urolithin metabolites. Fecal samples from the same subjects consuming walnut developed a different bacterial community structure compared to the baseline, indicating a potential impact of walnut diet on bacterial composition. A previous study [35] also reported gastrointestinal microbiota alterations, with increased relative abundance of *Faecalibacterium*, *Clostridium*, *Dialister*, and *Roseburia* after walnut consumption, but the link to individual urolithin metabolites was not clear [32, 36].

Our 16S RNA gene sequencing analyses of human gut microbiota revealed features that were associated with urolithin metabolites. We report that relative abundance of *Gordonibacter* was significantly enriched after walnut supplementation and significantly associated with urolithin A metabolites. *Gordonibacter* has been proposed to harbor metabolic capacity to transform ellagic acid into some intermediary urolithins in vitro [19, 31, 37]. For example, in vitro studies showed that *Gordonibacter* converted ellagic acid into urolithin C, which is then further metabolized to urolithin A or iso-urolithin A [19, 31]. Our observation that *Gordonibacter* abundance correlated with urolithin A production following walnut intake agrees with these previous reports. In addition to *Gordonibacter,* other bacteria are known to be involved in urolithin production, such as *Eggerthella* genera, which are key players in the metabolism of ellagitannins into urolithins, particularly urolithin A and iso-urolithin A [19]. However, we did not observe a significant enrichment in the relative abundance of *Eggerthella* after continuous walnut supplementation. This may be attributed to variations in an individuals' microbiota composition and competitive microbial dynamics within the gut. In particular, *Gordonibacter* may have out-competed *Eggerthella* for the available substrates provided by walnut, leading to its more prominent enrichment. Additionally, individual differences in baseline microbial populations could influence how specific bacteria respond to dietary interventions like walnut ingestion. These factors highlight the complex interactions that govern an individual's gut microbiota response to diet.

A study using a cohort of healthy Chinese youth exposed to high content ellagic acid delivered by capsules containing pomegranate seed extract also showed increased relative abundance of *Gordonibacter* and *Roseburia* [38]. Similarly, a relative increase in *Gordonibacter* was observed in a Spanish healthy cohort following short dietary walnut intake (3 days, 33 g/day) [32]. Consistent with these findings, our study reported that 9 bacterial genera (in a total of 13) positively correlated with iso-urolithin A metabolites, including *Roseburia*, and 4 bacterial genera (in a total of 4) positively correlated with urolithin A metabolites, including *Gordonibacter*, which significantly increased after walnut supplementation. Ultimately, the individual contribution of these microorganisms to the generation of the various urolithin metabolites will require isolation and in vitro characterization of their metabolic capacity.

A previous report identified bacterial pathways associated with ellagic acid metabolism and urolithin metabotypes (UMs) [38]. Interestingly, these KEGG orthologs did not overlap with the ones identified in our study. This discrepancy could be related to the grouping of urolithin metabolites into three different metabotypes rather than assessing correlations with individual metabolites. In our study, we only observed 2 KEGG orthologs, uridine diphosphate − N − acetylglucosamine (UDP-GlcNAc) kinase and adenine − specific DNA − methyltransferase (over a total of 7), that positively correlated with iso-urolithin A production. UDP-GlcNAc kinase phosphorylates UDP-GlcNAc, which is a precursor implicated in bacterial cell wall synthesis [39], whereas adenine − specific DNA − methyltransferase is a DNA adenine methylase (Dam) system whose activity impacts numerous biological processes, including DNA replication and transcription [40]. The functional relationship between these particular orthologs and walnut-derived ellagic acid metabolism is unclear. Future studies addressing changes to the bacterial metatranscriptome following a walnut enriched diet may provide further insight into gene pathways associated with urolithin metabolite production.

In summary, our study showed that the intestinal microbiota and the levels of urolithin metabolites are modified by walnut supplementation in healthy subjects, with an increased relative abundance of *Gordonibacter* genera associated with urolithin A. Since urolithin A concentrations in human serum and tissues depend upon gut microbial composition, further studies are needed to investigate the functional impact of bacterial genes on walnut metabolism and urolithin levels. Such studies may lead to the creation of specific bacterial designer consortium to augment the production of anti-inflammatory urolithins in humans.

## Supplementary Information

Supplementary Material 1.

Supplementary Material 2.

Supplementary Material 3.

Supplementary Material 4.

Supplementary Material 5.

Supplementary Material 6: Figure 1. Walnut consumption significantly augmented levels of urolithins. (A) Boxplot showing sum of normalized urolithin metabolites at both timepoints (*p*=0.00003). (B) PCA showing sample clustering based on their total urolithin metabolites (*p*=0.0000131). (C) Box plot showing individual unnormalized urolithin metabolite level of total urolithin metabolite (*p*=0.00089), urolithin A metabolite (*p*=0.00006), and iso-urolithin A metabolite (*p*=0.02204) before and after walnut dietary intervention. Samples before walnut dietary intervention represent timepoint A (*n*=38), and samples after walnut dietary intervention represent timepoint B (*n*=39). *P* values <0.05 were considered statistically significant.

Supplementary Material 7: Figure 2. Box plots for (A) PCoA1 and (B) PCoA2 show the difference between the two groups. Differences were tested using the Linear Mixed-Effects Model (lme) in R nlme package with the subject set as a random effect to account for multiple measures from the same subject.

## Data Availability

16S RNA gene sequencing reads have been deposited in the National Center for Biotechnology Information (NCBI) Sequence Read Archive (SRA) under bioproject ID: PRJNA1079311.
