# Supplementary figures and images for: Correlation between intestinal microbiota and urolithin metabolism in a human walnut dietary intervention
Source: BMC Microbiol. 2024 Nov 15;24:476. doi: 10.1186/s12866-024-03626-5 (PMC11566485; doi:10.1186/s12866-024-03626-5)

# Supplementary Figure 1

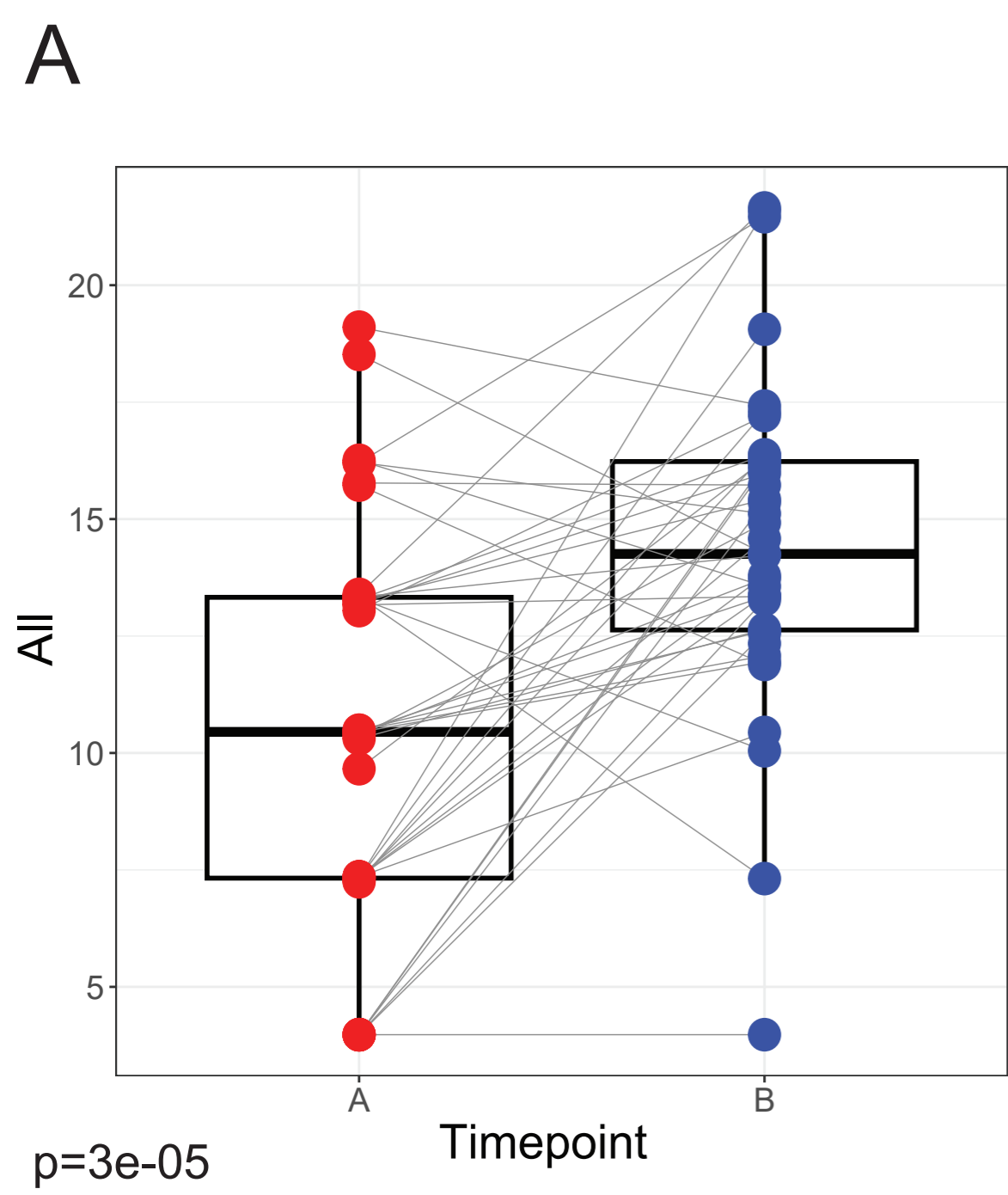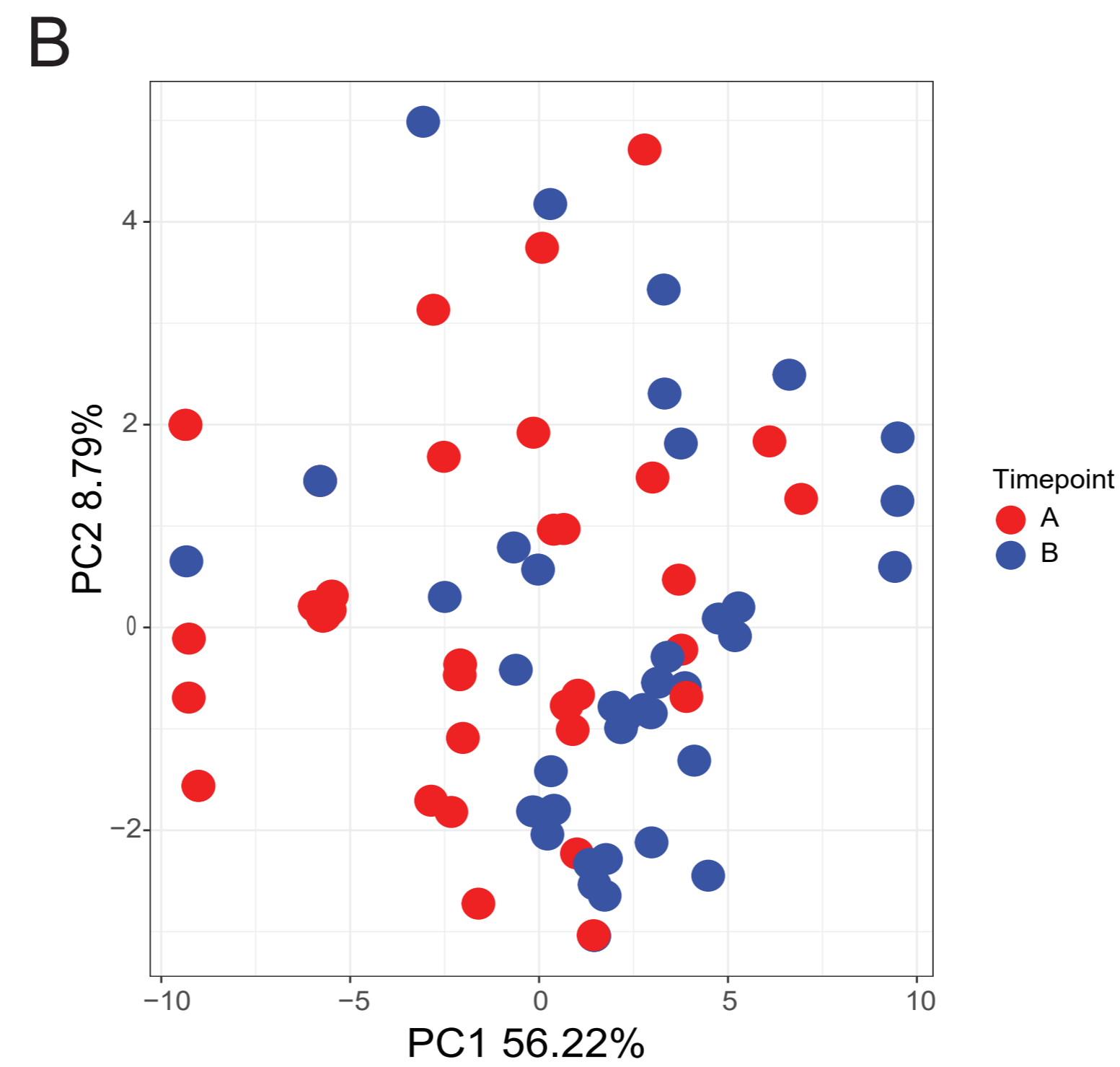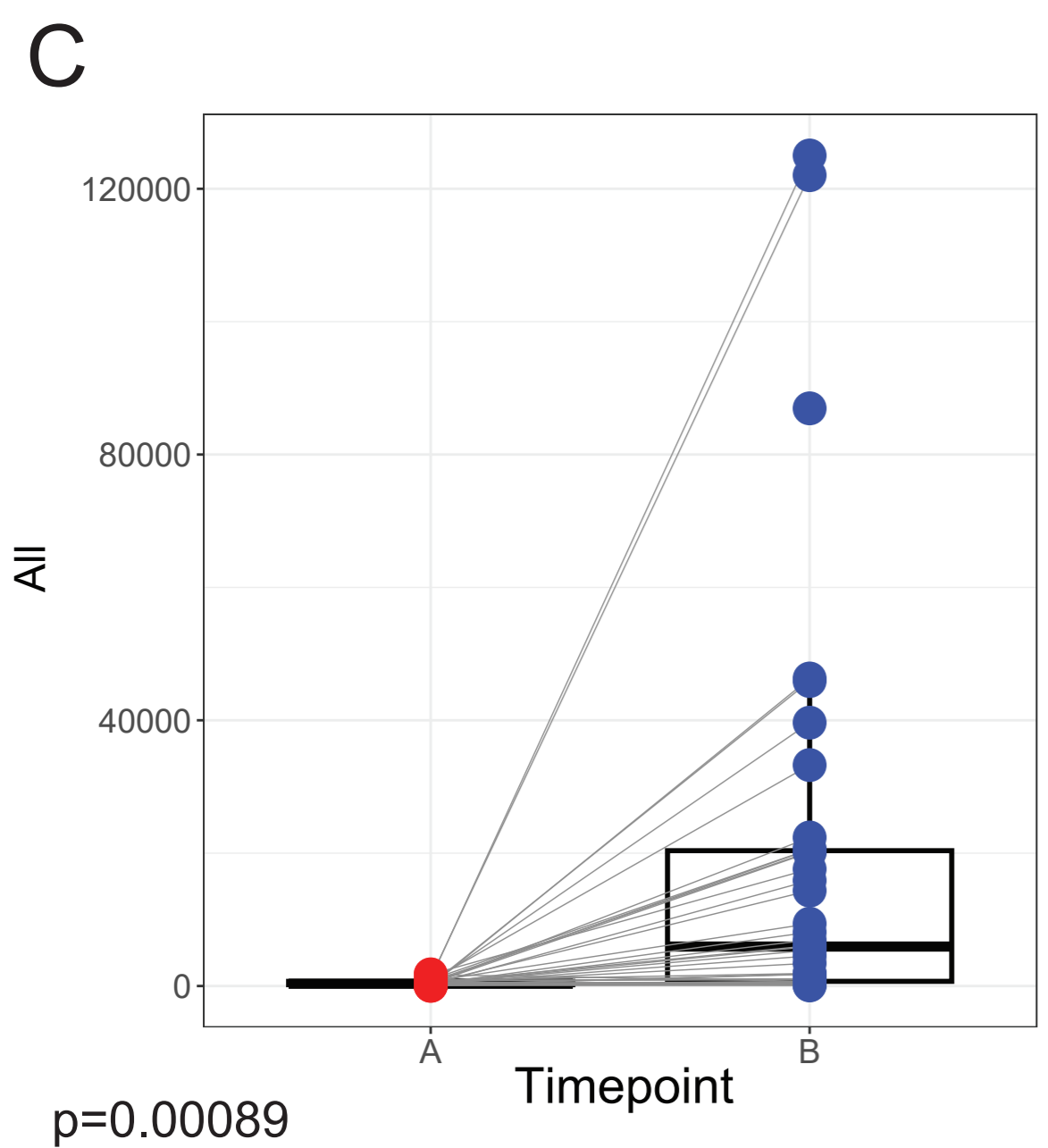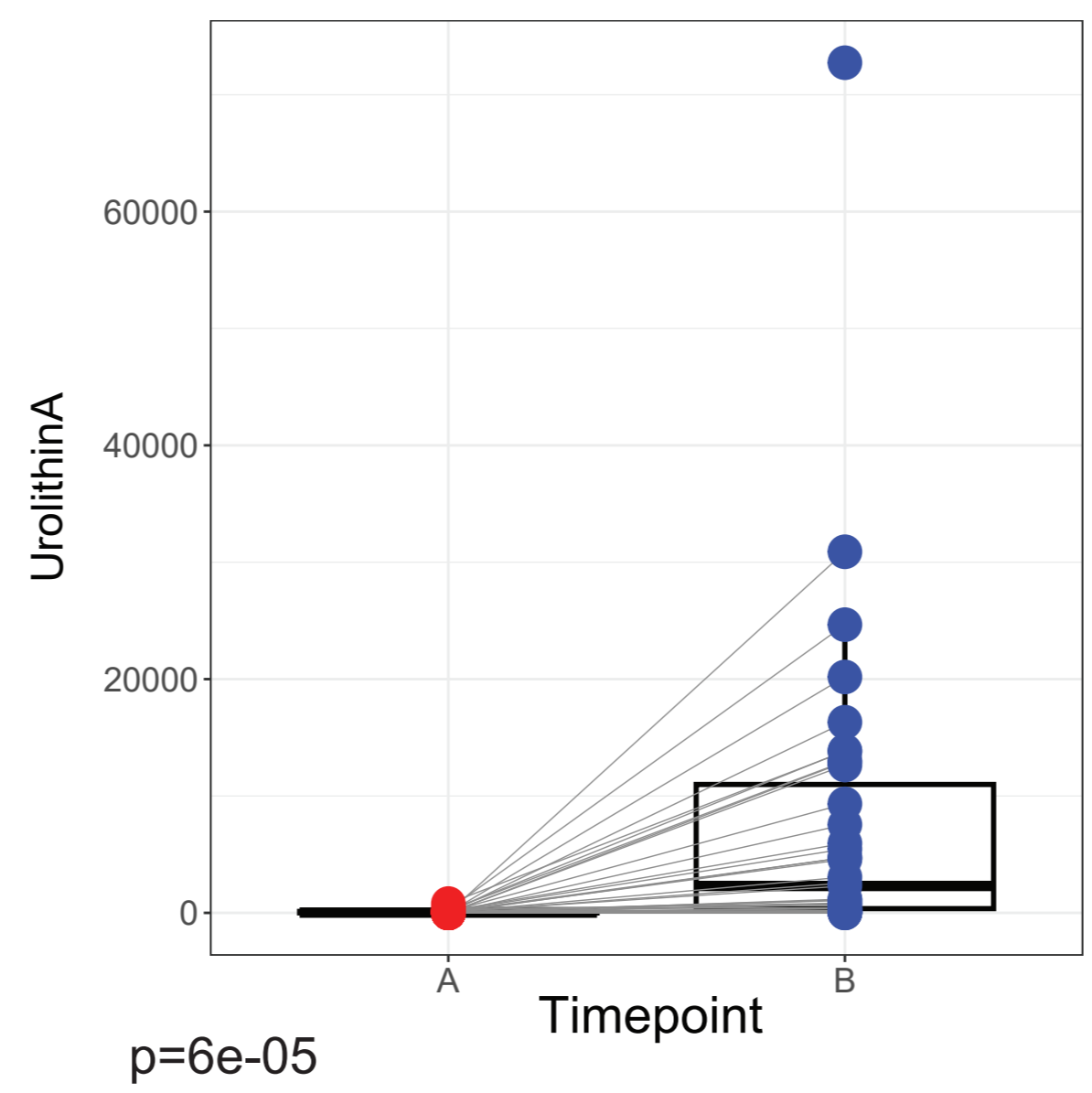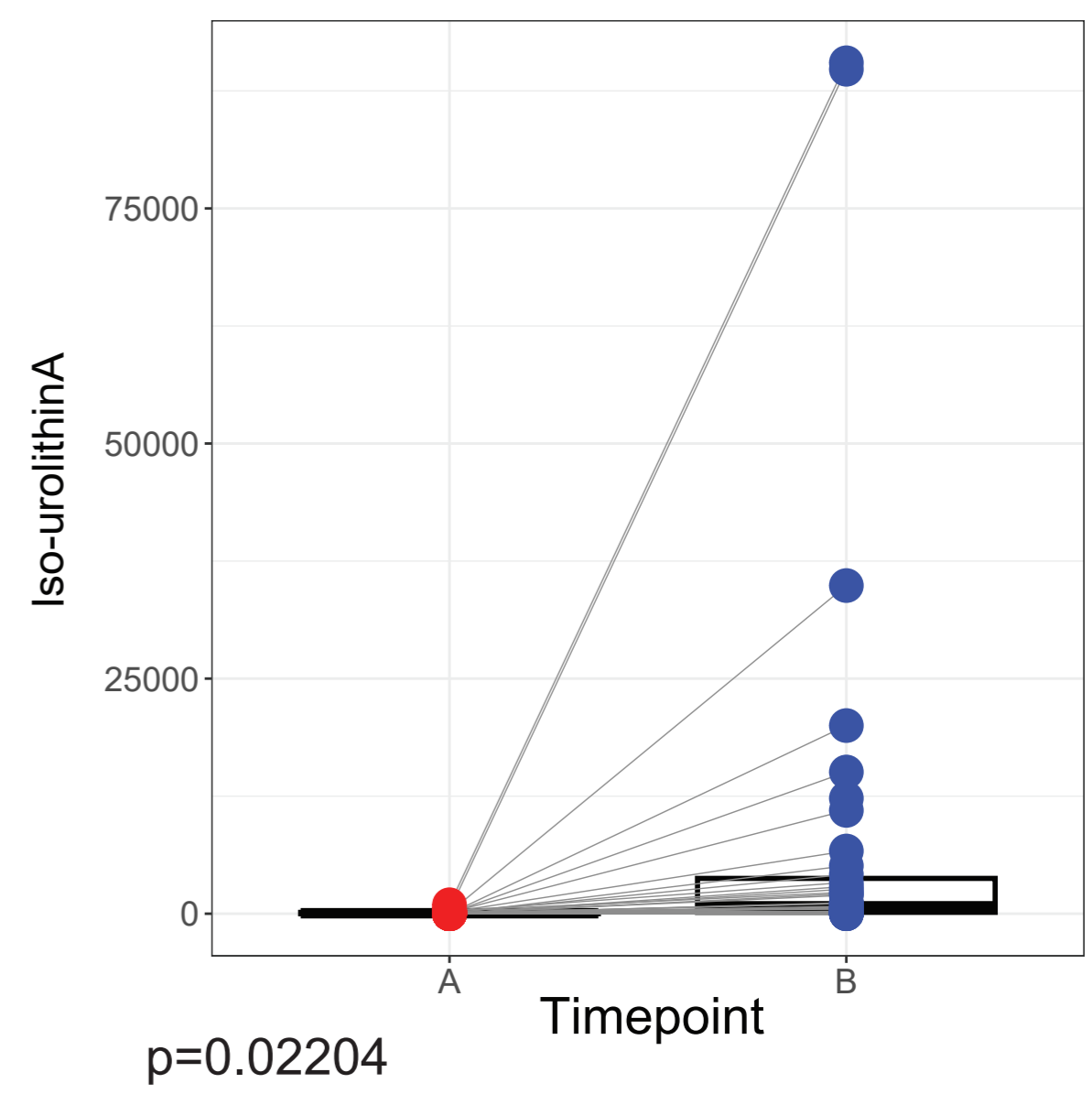

Supplement: Supplementary file 6 — Supplementary Material 6: Figure 1. Walnut consumption significantly augmented levels of urolithins. (A) Boxplot showing sum of normalized urolithin metabolites at both timepoints (p=0.00003). (B) PCA showing sample clustering based on their total urolithin metabolites (p=0.0000131). (C) Box plot showing individual unnormalized urolithin metabolite level of total urolithin metabolite (p=0.00089), urolithin A metabolite (p=0.00006), and iso-urolithin A metabolite (p=0.02204) before and after walnut dietary intervention. Samples before walnut dietary intervention represent timepoint A (n=38), and samples after walnut dietary intervention represent timepoint B (n=39). P values <0.05 were considered statistically significant. [file 12866_2024_3626_MOESM6_ESM.pdf]

# Supplementary Figure 2

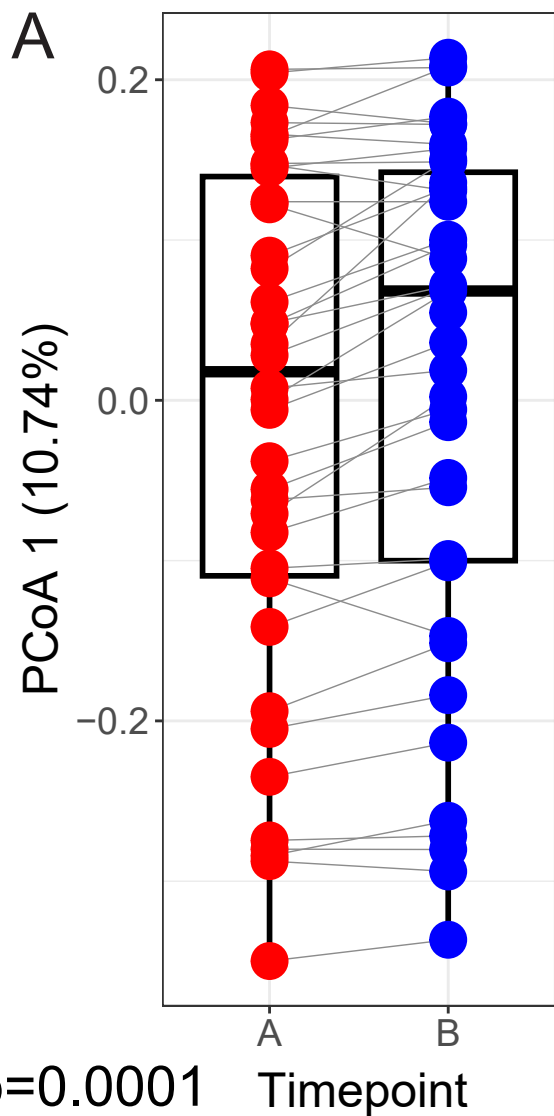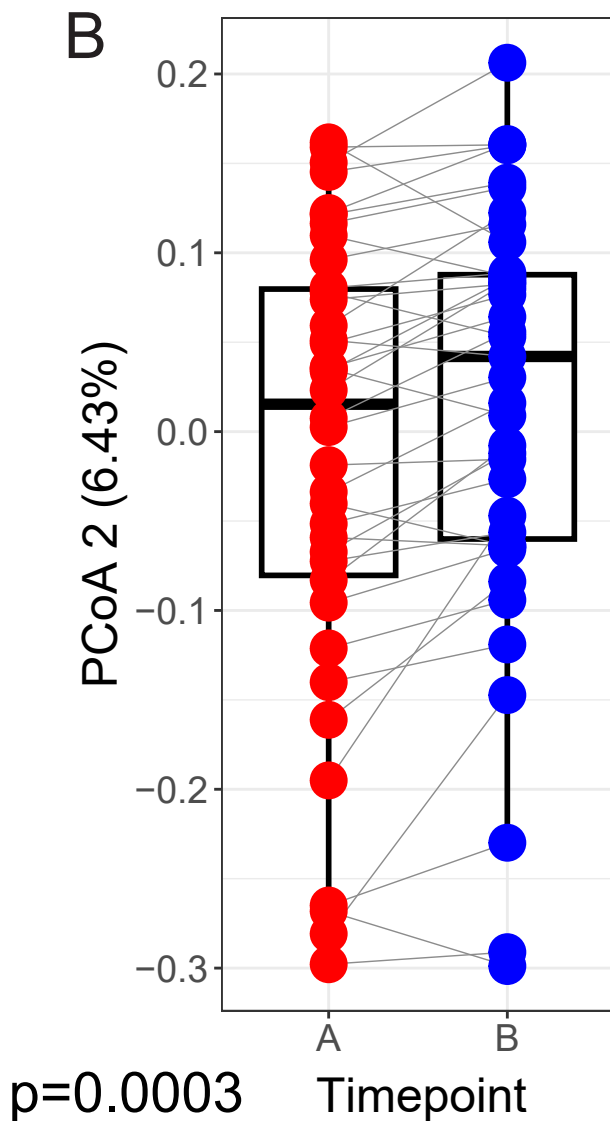

Supplement: Supplementary file 7 — Supplementary Material 7: Figure 2. Box plots for (A) PCoA1 and (B) PCoA2 show the difference between the two groups. Differences were tested using the Linear Mixed-Effects Model (lme) in R nlme package with the subject set as a random effect to account for multiple measures from the same subject. [file 12866_2024_3626_MOESM7_ESM.pdf]
